# Association between cannabis potency and mental health in adolescence

**DOI:** 10.1016/j.drugalcdep.2024.111359

**Published:** 2024-06-14

**Authors:** Lindsey A. Hines, Rebecca Cannings-John, Jemma Hawkins, Chris Bonell, Matthew Hickman, Stanley Zammit, Linda Adara, Julia Townson, James White

**Affiliations:** a Population Health Sciences, Bristol Medical School, University of Bristol, Bristol, UK; b Department of Psychology, University of Bath, Bath, UK; c Centre for Trials Research, School of Medicine, Cardiff University, Cardiff, UK; d DECIPHer, School of Social Sciences, Cardiff University, Cardiff, UK; e Department of Public Health, Environment and Society, London School of Hygiene & Tropical Medicine, London, UK; f MRC Centre for Neuropsychiatric Genetics and Genomics, Cardiff University, Cardiff, UK

**Keywords:** Cannabis, THC, Depression, Anxiety, Hallucination, Adolescent

## Abstract

**Introduction:**

In legal and illegal markets, high-potency cannabis (>10 % delta-9-tetrahydrocannabinol (THC)) is increasingly available. In adult samples higher-potency cannabis has been associated with mental health disorder but no studies have considered associations in adolescence.

**Methods:**

A population-wide study compared no, low and high potency cannabis using adolescents (aged 13–14 years) self-reported symptoms of probable depression, anxiety, and auditory hallucinations.

**Results:**

Of the 6672 participants, high-potency cannabis was used by 2.6 % (n=171) and low-potency by 0.6 % (n=38). After adjustment for sociodemographic factors, tobacco and alcohol use, in comparison to participants who had never used cannabis, people who had used high-potency but not low-potency cannabis were more likely to report symptoms of depression (odds ratio 1.59 [95 % confidence interval 1.06, 2.39), anxiety (OR 1.45, 95 % CI 0.96, 2.20), and auditory hallucinations (OR 1.56, 95 % CI 0.98, 2.47).

**Conclusions:**

High-potency cannabis use is associated with an increased risk of probable mental health disorders. Services and programming to minimise drug harms may need to be adapted to pay more attention to cannabis potency.

## 1 Introduction

Cannabis use policy is liberalising in many jurisdictions, and regular cannabis use has become more prevalent where cannabis is legal (United Nations: World Drug Report, 2023). Cannabis potency, defined as the concentration of Δ^9^-tetrahydrocannabinol (THC), doubled in the USA and Europe between 2008 and 2017 (Chandra et al., 2019; Petrilli et al., 2022), and new legal markets have facilitated the rapid development of cannabis products with higher potencies. Understanding the health effects of higher-potency cannabis is important as the experimental admiration of THC produces dose-dependent effects on anxiety and psychosis-like experiences (D’Souza et al., 2004), suggesting people who use higher-potency cannabis products may exposed to more risk.

A systematic review of 20 observational studies with adults found higher-potency cannabis was associated with increased risk of psychosis, and may be associated with anxiety, but little evidence was found for an association with depression (Petrilli et al., 2022). As far as we are aware, no studies have considered the relationship between cannabis potency and mental health in adolescence, despite evidence that the risks from cannabis use are greatest when use starts at younger ages (Hosseini and Oremus, 2019). Accordingly, we compared the mental health of people who use high- and low-potency cannabis in adolescence.

## 2 Materials and methods

### 2.1 Study sample

Data were from baseline assessments within a randomised controlled trial conducted in England and Wales (White, 2019). Schools were randomly sampled, and questionnaires completed before allocation. All procedures were approved by Cardiff University’s School of Social Sciences Ethics Committee (SREC/3342; 11th June 2019) and comply with the Helsinki Declaration of 1975, as revised in 2008. Written informed consent was obtained from all participants. The data that support the findings of this study are openly available at http://doi.org/10.17035/d.2023.0244798057. This manuscript adheres to the STROBE reporting guidelines (Von Elm et al., 2007).

### 2.2 Measures

*Probable depressive disorder* was assessed using 13-item Short Mood and Feelings Questionnaire applying the ≥12 cut-off point to indicate a disorder (Ancold and Stephen, 1995). *Probable anxiety disorders* were assessed using the Generalized Anxiety Disorder scale applying the ≥10 cut-off point (Spitzer et al., 2006). *Auditory hallucinations* were self-reported using three questions from the World Health Organization Composite International Diagnostic Interview (Kessler and Üstün, 2004).

*Cannabis potency* was assessed by asking participants whether they have ever tried “Cannabis (also called: marijuana, spliff, hash, skunk, grass, draw, dab, shatters)”. Those who reported having tried cannabis were asked “What type of cannabis have you most commonly used in the past year?” and were able to select from the following options: “herbal cannabis/ marijuana (herbal cannabis containing seeds, dark green to brown colour, earthy smell)”, “skunk/ sinsemilla/ other high-potency herbal cannabis (herbal cannabis without seeds, bright green, strong smell)”, “hashish/ resin/ solid (compressed blocks, brown coloured)”, “cannabis concentrate/ shatters (cannabis in a viscous oil or brittle form, extremely strong effects)”, or “I don’t know which type”. Images used in previous studies to estimate the relative concentrations of THC and cannabidiol (CBD) in each type of cannabis were provided to aid identification (see the Appendix; Freeman et al., 2014; Potter et al., 2018; Raber et al., 2015; Wilson et al., 2019). To be consistent with previous research that has validated self-reported data on three types of cannabis according to concentrations of THC and CBD among people who use cannabis in the UK (Freeman et al., 2014), we categorised use into never, used low (typically <10 % THC, 1–2 % CBD; “herbal cannabis/ marijuana”, “hashish/ resin/ solid”) or high-potency cannabis (typically >10 % THC, <1 % CBD; “skunk/ sinsemilla/ other high potency herbal cannabis”, “cannabis concentrate/ shatters”; Di Forti et al., 2019)).

Covariates were sociodemographic factors including self-reported gender identity (boy, girl, gender minority), age, ethnicity, socioeconomic disadvantage (free school meal entitlement, parental unemployment), smoking, alcohol consumption, frequency of cannabis use, and problematic cannabis use based on scoring ≥2 on the Cannabis Abuse Screening Test (Legleye et al., 2007).

### 2.3 Analysis

Missing data per variable ranged from 1.0 % to 11.4 % (Appendix Table 1). Missing data in all variables (exposures, outcomes, and covariates) were addressed through multiple imputation using chained equations and included a number of auxiliary variables to make the missing at random assumption more plausible. Each model included all variables, including the following auxiliary variables: age, ethnicity, and problematic cannabis use, and alcohol consumption in the past 30 days. Estimates were obtained by pooling results across 20 imputed datasets using the Rubin rules and assessment of Monte Carlo errors suggested this was a suitable number of imputations (White et al., 2011).

To test differences in the characteristics of participants by cannabis potency, we used logistic regression for binary exposures, multinomial for categorical exposures and linear regression for age (years). We estimated odds ratios (ORs) for the association between cannabis potency and outcomes using multilevel logistic regression (students nested within schools). These are reported alongside 95 % confidence intervals (CIs). The association between low- and high-potency cannabis compared to never having used cannabis was adjusted for the potential confounding effects of age, gender identity, socioeconomic disadvantage, then weekly smoking and alcohol use. Sensitivity analyses were conducted after excluding participants with any missing data, with lifetime cannabis use as the exposure, comparing low versus high potency cannabis use, then applying inverse-probability weighting by a propensity score.

## 3 Results

Of 7077 eligible to take part, 6672 participated (94.3 % response). Of these respondents, 60 (1.0 %) did not know what type of cannabis they had used, so were removed from the dataset. Of the 6612 participants, 0.6 % reported low-potency and 2.6 % high-potency cannabis use. Use of high-compared to low potency cannabis was more common in participants who smoked, drank alcohol, a gender minority, and came from a socioeconomically deprived household, but were no more likely to have a cannabis use problem, or have used cannabis in the past month (Table 1).

People who used high-potency cannabis were more than three times as likely to report a probable depressive disorder, anxiety disorder and auditory hallucinations, compared to those who had never used cannabis (Table 2). There was evidence of similar associations in unadjusted analyses for people who used low-potency cannabis. There was little evidence of attenuation of these associations after adjustment for sociodemographic factors. Associations were, however, markedly reduced after the addition of weekly smoking and after the addition of alcohol use associations for low potency cannabis were no longer evident.

In the datasets where there was no missing data, the confidence intervals for estimates overlapped with those from the main results using imputed data indicating there were no meaningful differences. There was evidence of an association between lifetime cannabis use and symptoms of a depressive, anxiety, conduct disorder and auditory hallucinations. There was little difference between the estimates from the multivariate and propensity-score model (Appendix, Tables 2–5).

## 4 Discussion

To our knowledge, the present study is the first to report on the use of low- and high- cannabis potency in early adolescence. High-potency cannabis using adolescents were significantly more likely than their peers who had never used cannabis use to report a probable depressive disorder, anxiety disorder, and auditory hallucination.

Of those using cannabis, the majority (82 %) reported that they were using high-potency cannabis. In other general population samples where use of high-potency herbal cannabis was measured, prevalence of use was reported at 69 % in a global, self-selecting sample young adults who use drugs (Chan et al., 2017), and 13 % (Hines et al., 2020) in a UK general population sample of 24 year-old participants. In this context, the prevalence of high-potency cannabis use in this young adolescent sample is high. However, published analyses of police seizure data in the UK identified that 80 % of samples of herbal cannabis had high (≥10 %) THC content (Potter et al., 2018). Consequently the prevalence of use in the present sample may reflect the availability of high-potency cannabis in the UK’s illegal market.

The present study is the first to show associations between high potency cannabis use and mental health disorder apparent in adults are also evident in adolescence. We found associations between the use of high-potency cannabis and depression, anxiety, and auditory hallucinations, after adjustment for sociodemographic factors, tobacco and alcohol use. This is in line with existing research on high potency cannabis use and anxiety in older populations (Hines et al., 2020) and psychosis in adults (Chan et al., 2017; Di Forti et al., 2015), as well as research showing lower-potency cannabis is not associated with auditory hallucinations (Di Forti et al., 2019); but contrasts with studies that have not previously found cannabis potency is associated with depressive symptoms (Chan et al., 2017; Hines et al., 2020; Prince and Conner, 2019).

This study is the first to describe the use of high-potency cannabis in a general-population sample of adolescents, utilising validated measures of mental health and images to aid the identification of different types of cannabis. However, there are a number of limitations that need to be considered when interpreting the results of this work. Firstly, the study is cross-sectional and, consequently, it is not possible that reverse causation may explain our findings. For the mental health disorders we assessed symptoms are likely to fist emerging at a similar time to or after onset of cannabis use. Secondly, we cannot be certain participants are correct when stating the type of cannabis that they’re using. Thirdly, most of the sample did not report cannabis use and consequently some of the analyses might be underpowered.

## 5 Conclusions

Among early adolescents using cannabis in an illegal market, high-potency cannabis is the most used type of cannabis. Even in such a young population, associations with negative mental health are already present, and notably high-potency cannabis is most prevalent among groups which are already disadvantaged. Our findings signal to educators and clinicians a need for policies, organisational practices, and programmes that better attend to the potency cannabis in efforts to minimise harms associated with its use.

## Supplementary Material

Supplementary data associated with this article can be found in the online version at 10.1016/j.drugalcdep.2024.111359.

Supplementary text

## Figures and Tables

**Table 1:** Characteristics of young people by cannabis potency (n = 6612).

Characteristic	Cannabis use %			
	Never used (n = 6402)	Low potency (n = 38)	High potency (n = 171)^a^	*P* value for trend^b^
**Mental health outcomes**				
Probable depressive disorder^c^	21.5	35.6	50.2	<0.001
Probable generalized anxiety disorder^c^	18.9	38.8	41.7	<0.001
Auditory hallucination	18.7	25.9	44.8	<0.001
**Sociodemographic Characteristics**				
Gender identity				
Boy	51.3	63.7	47.2	
Girl	46.6	33.1	46.6	0.89
Gender minority	2.1	3.2	6.2	0.001
Age (years) (Mean, SD)	13.69 (0.40)	13.75 (0.37)	13.76 (0.39)	0.003
Black, Asian and Minority Ethnicity	8.6	7.9	7.7	0.67
Entitled to free school meals	10.5	17.6	19.1	<0.001
Parent(s) / carer(s) unemployed	5.8	5.4	15.4	<0.001
**Substance use**				
Smoked in the past week	0.4	22.9	31.6	<0.001
Consumed alcohol in past 30 days	36.4	90.8	93.0	<0.001
Screened for a cannabis use problem	0.0	22.2	21.3	<0.001
Frequency of cannabis in past 12 months				
Weekly or more	0.0	18.0	18.1	<0.001
Monthly	0.0	82.0	81.8	<0.001

a All numbers estimated from imputed proportions.

b Determined by logistic, linear, or multinomial regression.

c Probable depressive disorder: Scoring ≥12 on Short mood and feelings questionnaire; Probable generalized anxiety disorder: Scoring ≥ 10 on Generalized Anxiety Disorder Scale; Cannabis use problem: Scoring ≥ on the Cannabis Abuse Screening Test.

**Table 2:** Association between the reported cannabis potency with probable mental health disorders and auditory hallucinations (n = 6612).^a^

	Odds ratio (95 % confidence interval)			
	Unadjusted	Adjusted for sociodemographic factors^b^	Adjusted for tobacco use^c^	Adjusted for alcohol and tobacco use^c^
**Probable depressive disorder**				
Never used	1.00 (reference)	-	-	-
Low potency	2.46 (1.13, 5.34)	2.45 (1.13, 5.32)	1.84 (0.81, 4.20)	1.18 (0.50, 2.76)
High potency	3.71 (2.63, 5.26)	3.53 (2.49, 5.01)	2.47 (1.66, 3.68)	1.59 (1.06, 2.39)
**Probable generalized anxiety disorder**				
Never used	1.00 (reference)	-	-	-
Low potency	3.43 (1.58, 7.45)	3.44 (1.58, 7.50)	2.69 (1.19, 6.10)	1.91 (0.83, 4.41)
High potency	3.01 (2.11, 4.29)	2.86 (2.00, 4.09)	2.04 (1.35, 3.09)	1.45 (0.96, 2.20)
**Auditory hallucinations** ^d^				
Never used	1.00 (reference)	-	-	-
Low potency	1.54 (0.59, 3.97)	1.54 (0.59, 4.01)	1.10 (0.40, 3.01)	0.79 (0.29, 2.17)
High potency	3.34 (2.25, 4.94)	3.13 (2.10, 4.66)	2.20 (1.39, 3.48)	1.56 (0.98, 2.47)

a All results estimated from imputed data. Multivariable model adjustment is incremental.

b Sociodemographic factors comprise age, gender identity, free school meal entitlement and parent/caregiver unemployment.

c Tobacco use assessed as weekly cigarette smoking and alcohol assessed as consuming alcohol in the past 30 days.

d Analytical n = 4813 as excludes those who responded that they preferred not to say or didn’t know whether they had hallucinated.
