# Supplementary material for: Association between cannabis potency and mental health in adolescence
Source: Drug Alcohol Depend. Author manuscript; Available in PMC 2026 Oct 5. (PMC7619506; doi:10.1016/j.drugalcdep.2024.111359)
Supplement: Supplementary file 1 [file EMS213679-supplement-Supplementary_text.docx]

**Appendix**

**Table 1.** Number and percentage of missing data for each variable

**Table 2.** Odds ratio (95% confidence interval) for association between cannabis potency with probable depressive disorder, generalised anxiety disorder and auditory hallucinations in complete case sample (n = 3447)

**Table 3**. Odds ratio (95% confidence interval) for association between low compared to high potency cannabis with probable depressive disorder, generalised anxiety disorder and auditory hallucinations (n = 209)

**Table 4.** Odds ratio (95% confidence interval) for association between lifetime cannabis use with probable depressive disorder, generalised anxiety disorder and auditory hallucinations (n = 6612)

**Table 5.** Odds ratio (95% confidence interval) for association between cannabis potency with study outcomes, comparing multivariable adjustment and inverse probability weighting by a propensity score (n = 6612)

Table 1. Number and percentage of missing data for each variable

| **Characteristic** | **Missing** | |
| --- | --- | --- |
|  | **N** | **%** |
| *Exposure* |  |  |
| Cannabis potency | 72 | 1.1 |
| *Covariates* |  |  |
| Gender identity | 67 | 1.0 |
| Age | 189 | 2.9 |
| Ethnicity | 87 | 1.3 |
| Free school meals entitlement | 80 | 1.2 |
| Parent(s) / carer(s) unemployment | 602 | 9.1 |
| Weekly smoking status | 550 | 8.3 |
| Alcohol consumed in past 30 days | 754 | 11.4 |
| Cannabis use problems | 70 | 1.1 |
| 12-month frequency of cannabis | 37 | 0.6 |
| *Outcomes* |  |  |
| Probable depressive disorder | 494 | 7.5 |
| Probable anxiety disorder | 597 | 9.0 |
| Auditory hallucinations | 292 | 4.4 |

Table 2. Odds ratio (95% confidence interval) for association between cannabis potency with probable depressive disorder, generalised anxiety disorder and auditory hallucinations in complete case sample (n = 3447)

|  | **Unadjusted** | **Adjusted for sociodemographic factors** ^b^ | **Adjusted for tobacco use** ^c^ | **Adjusted for alcohol and tobacco use** ^c^ |  |  |
| --- | --- | --- | --- | --- | --- | --- |
| **Probable depressive disorder** | | | | |  |  |
| **Never used** | 1.00 (reference) | - | - | - |  |  |
| **Low potency** | 2.09 (0.71, 6.12) | 1.85 (0.59, 5.78) | 1.29 (0.38, 4.40) | 0.90 (0.25, 3.22) |  |  |
| **High potency** | 4.28 (2.72, 6.72) | 4.09 (2.52, 6.64) | 2.74 (1.57, 4.79) | 1.66 (0.92, 2.97) |  |  |
|  |  |  |  |  | |  |
|  | **Probable generalised anxiety disorder** | | | |  |  |
| **Never used** | 1.00 (reference) | - | - | - |  |  |
| **Low potency** | 3.23 (1.16, 9.04) | 3.13 (1.07, 9.22) | 2.65 (0.86, 8.11) | 1.47 (0.43, 5.05) |  |  |
| **High potency** | 4.15 (2.63, 6.54) | 3.82 (2.35, 6.21) | 3.05 (1.75, 5.30) | 1.84 (1.02, 3.32) |  |  |
|  |  | | | | |  |
|  | **Auditory hallucinations** ^d^ | | | |  |  |
| **Never used** | 1.00 (reference) | - | - | - |  |  |
| **Low potency** | 1.30 (0.37, 4.61) | 1.13 (0.30, 4.17) | 0.82 (0.21, 3.16) | 0.35 (0.07, 1.74) |  |  |
| **High potency** | 4.53 (2.88, 7.11) | 4.04 (2.53, 6.44) | 2.80 (1.64, 4.80) | 1.73 (0.98, 3.07) |  |  |

^a^ All results estimated from imputed data. Multivariable model adjustment is incremental.

^b^ Sociodemographic factors comprise age, gender identity, free school meal entitlement and parent/caregiver unemployment.

^c^ Tobacco use assessed as weekly cigarette smoking and alcohol assessed as consuming alcohol in the past 30 days.

^d^ Analytical n = 4813 as excludes those who responded that they preferred not to say or didn’t know whether they had hallucinated.

Table 3. Odds ratio (95% confidence interval) for association between low compared to high potency cannabis with probable depressive disorder, generalised anxiety disorder, and auditory hallucinations (n = 209)

|  | **Unadjusted** | **Adjusted for sociodemographic factors** ^b^ | **Adjusted for tobacco use** ^c^ | **Adjusted for alcohol and tobacco use** ^c^ | **Adjusted for the frequency of cannabis use** | | |
| --- | --- | --- | --- | --- | --- | --- | --- |
| **Probable depressive disorder** | | | | | | | |
| **Low potency** | 1.00 (reference) | - | - | - | - | | |
| **High potency** | 1.82 (0.83, 4.00) | 1.44 (0.62, 3.34) | 1.35 (0.56, 3.28) | 1.35 (0.55, 3.33) | 1.36 (0.97, 1.51) | | |
|  |  |  |  |  | |  |  |
| **Probable generalised anxiety disorder** | | | | | | | |
| **Low potency** | 1.00 (reference) | - | - | - | - | | |
| **High potency** | 1.10 (0.50, 2.44) | 0.83 (0.35, 1.97) | 0.76 (0.31, 1.86) | 0.76 (0.31, 1.87) | 0.75 (0.30, 1.88) | | |
|  |  | | | | |  |  |
| **Auditory hallucinations** | | | | | | | |
| **Low potency** | 1.00 (reference) | - | - | - | - | | |
| **High potency** | 2.26 (0.83, 6.16) | 2.03 (0.72, 5.71) | 2.01 (0.69, 5.84) | 1.98 (0.68, 5.76) | 1.97 (0.68, 5.75) | | |

^a^ All results estimated from imputed data. Multivariable model adjustment is incremental.

^b^ Sociodemographic factors comprise age, gender identity, free school meal entitlement and parent/caregiver unemployment.

^c^ Tobacco use assessed as weekly cigarette smoking and alcohol assessed as consuming alcohol in the past 30 days.

Table 4. Odds ratio (95% confidence interval) for association between lifetime cannabis use with probable depressive disorder, generalised anxiety disorder, and auditory hallucinations (n = 6612) ^a^

|  | **Unadjusted** | **Adjusted for sociodemographic factors** ^b^ | **Adjusted for tobacco use** ^c^ | **Adjusted for alcohol and tobacco use** ^c^ |  | |
| --- | --- | --- | --- | --- | --- | --- |
| **Probable depressive disorder** | | | | | |  |
| **Never used** | 1.00 (reference) | - | - | - | |  |
| **Lifetime user** | 3.12 (2.34, 4.15) | 3.23 (2.38, 4.38) | 2.33 (1.64, 3.31) | 1.51 (1.06, 2.17) | |  |
|  |  |  |  |  |  | |
| **Probable generalised anxiety disorder** | | | | | |  |
| **Never used** | 1.00 (reference) | - | - | - | |  |
| **Lifetime user** | 2.81 (2.10, 3.75) | 2.80 (2.06, 3.82) | 2.07 (1.44, 2.97) | 1.47 (1.02, 2.12) | |  |
|  |  | | | |  | |
| **Auditory hallucinations** ^d^ | | | | | |  |
| **Never used** | 1.00 (reference) | - | - | - | |  |
| **Lifetime user** | 3.15 (2.30, 4.32) | 2.83 (2.04, 3.91) | 2.16 (1.48, 3.14) | 1.50 (1.02, 2.20) | |  |

^a^ All results estimated from imputed data. Multivariable model adjustment is incremental.

^b^ Sociodemographic factors comprise age, gender identity, free school meal entitlement and parent/caregiver unemployment.

^c^ Tobacco use assessed as weekly cigarette smoking and alcohol assessed as consuming alcohol in the past 30 days.

^d^ Analytical n = 4813 as excludes those who responded that they preferred not to say or didn’t know whether they had hallucinated.

Table 5. Odds ratio (95% confidence interval) for association between cannabis potency with study outcomes, comparing multivariable adjustment and inverse probability weighting by a propensity score (n = 6612) ^a^

|  | **Multivariable adjustment** ^b^ | **Inverse probability weighting**  **by propensity score ^c^** |
| --- | --- | --- |
| **Probable depressive disorder** | | |
| **Never** | 1.00 (reference) | - |
| **Low potency** | 1.18 (0.50, 2.76) | 1.05 (0.89, 1.25) |
| **High potency** | 1.59 (1.06, 2.39) | 1.12 (1.01, 1.24) |
|  |  |  |
| **Probable generalized anxiety disorder** | | |
| **Never** | 1.00 (reference) | - |
| **Low potency** | 1.91 (0.83, 4.41) | 1.14 (0.98, 1.33) |
| **High potency** | 1.45 (0.96, 2.20) | 1.11 (1.00, 1.22) |
| **Auditory hallucinations** ^d^ | | |
| **Never** | 1.00 (reference) | - |
| **Low potency** | 1.54 (0.59, 3.97) | 0.96 (0.81, 1.14) |
| **High potency** | 3.34 (2.25, 4.94) | 1.12 (0.99, 1.27) |

^a^ All results estimated from imputed data.

^b^ Multivariable models adjusted for age, gender identity, free school meal entitlement, parent/caregiver unemployment, weekly cigarette smoking, and consuming alcohol in the past 30 days.

^c^ Propensity score estimated using logistic regression with the teffects ipwra command in Stata v17.0. Covariates included were from multivariable model 3: age, gender identity, free school meal entitlement, parent/caregiver unemployment, weekly cigarette smoking and consuming alcohol in the past 30 days.

^d^ Analytical n = 4813 as excludes those who responded that they preferred not to say or didn’t know whether they had hallucinated.

Cannabis potency questions

What type of **cannabis** have you most commonly used **in the past year**?

Look at the picture on the right to help with your answer

(Please mark an **X** in **ONE** box only)

Example **A** -**Skunk/Sinsemilla/other high potency herbal cannabis** (herbal cannabis without seeds, bright green, strong smell)

Example **B** - **Herbal cannabis/marijuana** (herbal cannabis containing seeds, dark green to brown colour, earthy smell)

Example **C** - **Hashish/resin/solid** (compressed blocks, brown coloured)

Example **D** - **Cannabis Concentrate/Shatters** (cannabis in a viscous oil or brittle form, extremely strong effects)

I **don’t know** which type

Reproduced with permission from David Potter and Tom Freeman
